# Machine Learning Analysis of the Anatomical Parameters of the Upper Airway Morphology: A Retrospective Study from Cone-Beam CT Examinations in a French Population

**DOI:** 10.3390/jcm12010084

**Published:** 2022-12-22

**Authors:** Caroline de Bataille, David Bernard, Jean Dumoncel, Frédéric Vaysse, Sylvain Cussat-Blanc, Norbert Telmon, Delphine Maret, Paul Monsarrat

**Affiliations:** 1 Laboratoire Centre d’Anthropobiologie et de Génomique de Toulouse, Université Paul Sabatier, 31073 Toulouse, France; 2 School of Dental Medicine and CHU de Toulouse—Toulouse Institute of Oral Medicine and Science, 31062 Toulouse, France; 3 Institute of Research in Informatics (IRIT) of Toulouse, CNRS—UMR5505, 31062 Toulouse, France; 4 RESTORE Research Center, Department of Oral Medicine, Université de Toulouse, INSERM, CNRS, EFS, ENVT, Université P. Sabatier, Toulouse University Hospital (CHU), Batiment INCERE, 4bis Avenue Hubert Curien, 31100 Toulouse, France; 5 Artificial and Natural Intelligence Toulouse Institute ANITI, 31013 Toulouse, France; 6 Service de Médecine Légale, Centre Hospitalier Universitaire Rangueil, Avenue du Professeur Jean Poulhès, CEDEX 9, 31059 Toulouse, France

**Keywords:** cone-beam CT, upper airway, anatomical parameters, soft tissues, machine learning, symbolic regression

## Abstract

The objective of this study is to assess, using cone-beam CT (CBCT) examinations, the correlation between hard and soft anatomical parameters and their impact on the characteristics of the upper airway using symbolic regression as a machine learning strategy. **Methods:** On each CBCT, the upper airway was segmented, and 24 anatomical landmarks were positioned to obtain six angles and 19 distances. Some anatomical landmarks were related to soft tissues and others were related to hard tissues. To explore which variables were the most influential to explain the morphology of the upper airway, principal component and symbolic regression analyses were conducted. **Results:** In total, 60 CBCT were analyzed from subjects with a mean age of 39.5 ± 13.5 years. The intra-observer reproducibility for each variable was between good and excellent. The horizontal soft palate measure mostly contributed to the reduction of the airway volume and minimal section area with a variable importance of around 50%. The tongue and the position of the hyoid bone were also linked to the upper airway morphology. For hard anatomical structures, the anteroposterior position of the mandible and the maxilla had some influence. **Conclusions:** Although the volume of the airway is not accessible on all CBCT scans performed by dental practitioners, this study demonstrates that a small number of anatomical elements may be markers of the reduction of the upper airway with, potentially, an increased risk of obstructive sleep apnea. This could help the dentist refer the patient to a suitable physician.

## 1. Introduction

Cone-beam computed tomography (CBCT) is an imaging technology that has been increasingly used in the last decade. During the procedure, the CBCT rotates around the patient’s head, using a cone-shaped beam to obtain many two-dimensional images. The scanning software reconstructs the data to produce values on a regular grid in three-dimensional space, and these values can be manipulated and visualized with specialized software [1]. Due to its high spatial resolution, isotropic voxel, adequate contrast between the soft tissue and empty space, and the relatively low radiation dose compared to conventional CT, CBCT can be used to analyze the anatomical parameters three-dimensionally [1]. CBCT is well suited for imaging the craniofacial area [1,2]. Depending on the size of the acquisition field, CBCT can show the soft and hard anatomical structures such as the maxillo-facial region, the base of the skull, the viscerocranium and the upper airway [1,2]. CBCT is consequently a tool for the diagnosis, assessment, planning, and delivery of treatment in various specialties in dentistry (e.g., endodontics, periodontology, orthodontics, oral and maxillofacial surgery and implantology, temporomandibular joint disorders) [3].

Compared with multidetector computed tomography (MDCT), CBCT is less costly, uses lower radiation doses for a higher resolution, and produces more detailed images of hard tissues, even if it provides less soft tissue contrast. Consequently, CBCT imaging could replace MDCT in otolaryngology-related applications, for instance for the evaluation of the upper airway, at standard or low-dose protocols [4,5].

The upper airway comprises the nasopharynx, the oropharynx, and the hypopharynx. Several hard and soft tissue particularities constrain the aerial flow. According to the literature, CBCT is an accurate tool for upper airway evaluation [6,7,8,9,10,11,12,13,14,15,16]. Indeed, CBCT provides insights into the anatomical anomalies found along craniofacial structures and has been used to measure soft and hard anatomical structures. It has been suggested that anatomical abnormalities of the upper airway, including upper-airway collapsibility, alterations in craniofacial structures (i.e., the anteroposterior position of the mandible), and enlargements of surrounding soft tissue structures (i.e., tongue and lateral pharyngeal walls, soft palate, and hyoid bone) play an important role in the development of Obstructive Sleep Apnea (OSA) [6,7,8,9,10,11,12,13,14,15,16]. This pathology is characterized by the temporary cessation of breathing (apnea) or shallow breathing (hypopnea) with decreased hemoglobin oxygen saturation [9,10,11,16]. Increased interest in upper airway morphology can be attributed to the appreciation that the upper airway configuration is associated with OSA as well as its general relationship to craniofacial morphology.

Although several studies using CBCT have been carried out to determine the upper-airway configuration, they used various anatomical structures without investigating the role of all these structures taken simultaneously [6,7,8,9,10,11,12,13,14,15,16]. In the literature, anatomical landmarks seem easily identifiable without superimposition or distortion on CBCT exams [17,18,19,20,21,22,23,24,25,26,27,28,29]. The upper airway volume can be obtained by the automatic segmentation method [8,9,13]. It would be interesting to analyze the different variables and see how they are linked to each other, specifically exploring the placement of variables related to the upper airway. Moreover, in recent years, the use of Machine Learning (ML) methods has made it possible to accurately estimate the observed dynamics by learning automatically from several variables. In this study, in addition to the classical statistical approach, we use a special branch of ML, namely symbolic regression (SR). SR is a type of regression analysis that searches the space of mathematical expressions to find the model that best fits data. SR works by randomly combining mathematical building blocks to obtain models understandable from a human perspective.

The aim of this study was to assess on CBCT exams the correlation between hard and soft anatomical parameters and their impact on the characteristics of the upper airway using symbolic regression as a machine learning strategy.

## 2. Materials and Methods

### 2.1. Sample

All CBCT scans were performed by an experienced dentist (EC), specialized in oral and maxillo-facial radiology for more than 10 years. All scans were anonymized before being transmitted for analysis. All CBCT scans performed in 2015 were considered for inclusion.

Inclusion criteria were as follows: adult subjects who had finished their growth (age up to 22 years); subjects with bilateral occlusal contacts; CBCT acquisitions had to cover the entire upper airway, from the hard palate to the lowest anterior point of the 3rd cervical vertebral body. Non-inclusion criteria were as follows: poor quality of scans (motion or metallic artefacts resulting in a significant impact on the usability of the exam); lack of at least one anatomical landmark (Table 1).

### 2.2. Data Acquisition

All CBCT examinations were performed by a CBCT scanner (CS 9500 3D^®^, Carestream, Marne-la-Vallée, France) with tube voltage of 90 kV and tube current of 10 mA. The voxel size was 300 µm and the FOV was 90 × 150 mm. The exposure time was 10.8 s with a dose–area product of 605 mGy·cm${}^{2}$. The scans were acquired according to the manufacturer’s recommended protocol with the minimum exposure necessary for adequate image quality (ALADA principles, “As Low as Diagnostically Acceptable”). No CBCT examination was performed specially for the study (medical reasons only). During image acquisition, the patient was positioned upright. The CBCT images were exported as DICOM (.dcm) files and then imported into the software program Avizo 8.1 (Thermo Fischer Scientific, Villebon, France) for analysis.

### 2.3. Radiographic Analysis

The protocol for the automatic segmentation and landmarks positioning is provided in Supplementary Text.

### 2.4. Volume Reorientation

Three anatomical landmarks (rPo, rOr and lOr (Table 1)) were positioned to identify the Frankfort horizontal plane (FH plane), and the radiological volume was reoriented with respect to this plane. Then, three other anatomical landmarks (Na, ANS and MGNM, Table 1) were positioned to identify the midsagittal plane; the radiological volume was then reoriented with respect to this plane. Finally, each CBCT exam was reoriented with respect to the FH and the midsagittal plane.

### 2.5. Volume Segmentation, Volume, and Minimal Cross-Sectional Area (CSAmin)

The upper airway was segmented using the Avizo^®^ software. After thresholding to distinguish hard/soft tissues from aerial cavities, the volume of the upper airway was automatically segmented between the superior boundary (i.e., the plane going through PNS and ANS, parallel to FH plane), and the inferior boundary (i.e., the plane going across the anteroinferior point of the body of the 3rd cervical spinal vertebra, parallel to the FH plane). The volume was then determined by using the Avizo “measure and analysis” tool. The minimal cross-sectional area (CSAmin) was defined as the slice of the upper airway with the minimal area. The anteroposterior and lateral dimensions of the CSAmin were then measured by using the Avizo 3D linear measuring tool (Ap and Lat, respectively, as shown in Table 2).

### 2.6. Landmarks

Twenty-four anatomic landmarks were positioned within each CBCT exam (Table 1) using the axial, sagittal, coronal planes and the Avizo “Isosurface” tool. Once the landmark coordinates were exported into ASCII files, mathematical formulae were applied to obtain the 6 angles and 19 distances presented in Table 3 (we used a script operated with Scilab 6.0.1). Some anatomical landmarks are related to soft tissues and others are related to hard tissues.

### 2.7. Statistical Analyses

*Reproducibility of measurements:* Intra-examiner reproducibility at 1-week interval was assessed computing intraclass correlation coefficients (ICC) [30]. Only distances, angles, area, and volumes were considered for reproducibility analysis; reproducibility of the landmarks positioning per se was not considered.

*Data analysis:* Each outcome was firstly described using means, standard deviations, and quartiles. Quartiles were added to the descriptive statistics, since most of the variables did not meet the criteria of normality (as assessed by the Shapiro–Wilk test for normality). The correlations between the variables were computed two by two using the Kendall’s Tau correlation coefficient. The level of significance was set at 5% (*p* < 0.05). For multivariate analysis, two approaches were considered. (1) A principal component analysis (PCA) was performed to see how the different variables were related to each other in the different dimensions, exploring the positioning of the variables related to the upper airway. The R packages “FactoMineR” and “factoextra” were used. (2) To explore which variables were the most influential to explain the upper airway volume and the CSAmin, symbolic regression analyses were performed using Eureqa software 1.24.0 (Nutonian, Boston, MA, USA). Ten independent experiments were run for both the minimum cross-sectional area and the upper airway volume with absolute error as fitness metric, 80% of the dataset being randomly affected to training, and 20% being dedicated to model validation. The following mathematical operations (i.e., building blocks) were allowed: addition, subtraction, multiplication, division, exponential natural logarithm, power, and square root. A minimum of $10^{11}$ formula evaluations and $2\times 10^{6}$ generations were performed for each run. The normalized fitness-weighted variable importance was then computed as defined by Vladislavleva et al. [31]. The mean magnitude of effects for each contributing variable was computed from the best model of each experiment. The magnitude of effects (sensitivity analysis) means that when the variable increases, there is an increase (positive magnitude) or a decrease (negative magnitude) in the target variable (Vol, CSAmin). All details for computing normalized fitness-weighted variable importance and magnitude of effects are provided in the Supplementary Text.

## 3. Results

### 3.1. Study Sample

Overall, 64 CBCT were considered for potential inclusion, of which CBCT scans were excluded because of metallic and motion artefacts. A total of 60 CBCT scans were considered in this retrospective study. The mean age of our study subjects was 39.5 ± 13.5 years, and they were predominantly women (42 women, 70%).

### 3.2. Reproducibility of Measurements

ICCs for intra-observer assessments for each outcome are shown in Table 4. Values were almost superior to 0.7 and are considered as good to excellent. The lowest reliability was obtained for the SNA angle (0.67 [0.34; 0.86]).

### 3.3. Descriptive Analysis

Table 5 presents the descriptive characteristics of the upper airway for each measured outcome. A particularly striking element is the wide variability in the upper airway measures (14,462 ± 7399 mm${}^{3}$ for the volume, 206 ± 123 mm${}^{2}$ for the CSAmin). Our subjects preponderantly exhibit a maxillary retroposition relative to the cranial basis (mean SNA of 81.8 ± 4.4), a mandibular retroposition (mean SNB of 77.8 ± 5.0), and a mid-facial morphotype (mean FMA of 33.3 ± 6.5) [32].

### 3.4. Bi-Variate Analysis

**Figure 1:**
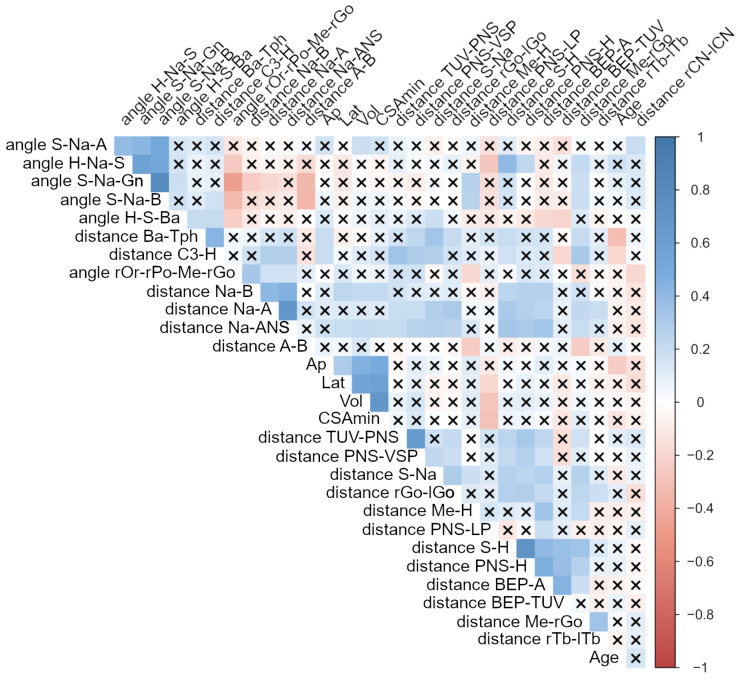
The correlation matrix plot between the variables two by two using Kendall’s τ correlation coefficient. The level of significance was set at 5% (*p* < 0.05). The colormap indicates the values of the correlation (from strong positive correlation in deep blue to strong negative correlation in deep red). A cross means non-significance at the 5% threshold.

The correlation matrix was presented in Figure 1. The anteroposterior position of the mandible (Na–B distance) was significantly correlated with the characteristics of the upper airway (volume, CSAmin and its anteroposterior and lateral dimension), the hyoid bone position (S–H distance), the position of the tongue (BEP–A distance) and the anteroposterior position of the maxilla, with τ correlation coefficients around +0.4.

The height of the nasal cavity (Na-ANS distance) was slightly but significantly correlated with the characteristics of the upper airway and the localization of the soft palate (TUV–PNS) (τ around +0.2). It was moderately associated with the dimension of the tongue (BEP–A), the width of the mandible (rGo–lGo), the dimension of the cranial basis (S–Na), the position of hyoid bone (S–H, PNS–H), the dimension of vertical soft palate (PNS–VSP) and the anteroposterior position of the maxilla (Na–A), with τ values around +0.4.

The horizontal soft palate (PNS-LP) was significantly correlated with the characteristic of upper airway with τ correlation coefficients around −0.3, and it was moderately correlated with the dimension of the tongue (BEP-A) with τ values around +0.4. Furthermore, age was significantly correlated with τ values around +0.4 with the anteroposterior dimension of the CSAmin, the pharyngeal hypertrophy (Ba-Tph) and the hyoid bone position (C3–H). There was a negative correlation between these two variables (τ = −0.3, *p* < 0.05).

### 3.5. Results from the Principal Component Analysis

**Figure 2:**
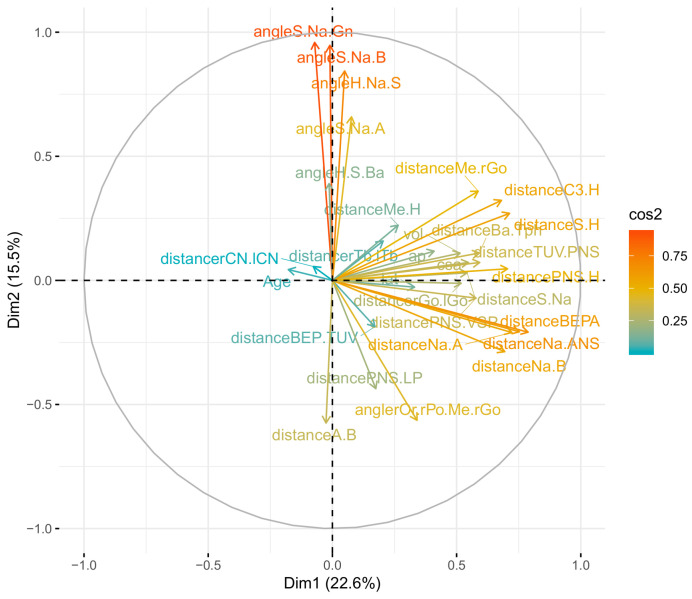
Results of the principal component analysis (PCA) for the dimensions 1 and 2. Variables well represented by the PCA are located next to the periphery of the circle (and have a high value of cos2). Positively correlated variables are found closely, while negatively correlated variables are found in the opposite quadrant.

This analysis highlights the most important variables which are associated in each dimension to explain the variability of the sample subjects. Most of the variability was explained by the first two dimensions, with 22.6% and 15.5% of the explained variance, respectively (Figure 2). We observed that the variables which were the most correlated to the upper airway characteristics were: for soft tissues, the dimension of the tongue (BEP–A), the hyoid bone position (C3–H, S–H), pharyngeal hypertrophy (Ba–Tph), and the soft palate (PNS–LP, PNS–VSP); for hard tissues, the position of the anteroposterior mandible (Na–B), and the length of the nasal cavity (Na–ANS).

### 3.6. Machine Learning Analysis

**Figure 3:**
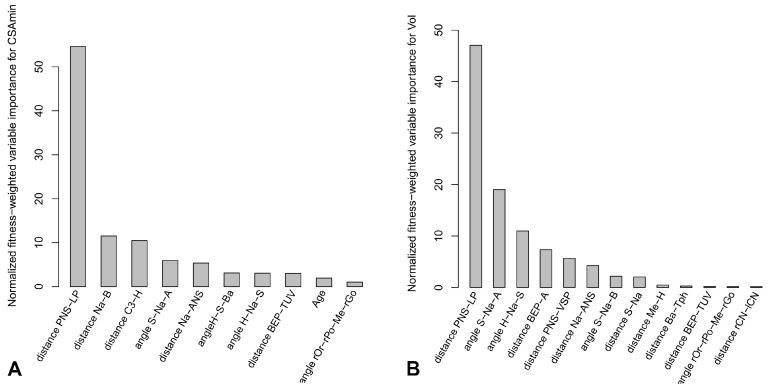
Results for the symbolic regression analysis. (**A**) The variables are ranked by decreasing normalized fitness-weighted variable importance to explain the CSAmin of the upper airway. (**B**) The variables are ranked by decreasing normalized fitness-weighted variable importance to explain the volume (Vol) of the upper airway.

Since numerous variables are highly correlated or weakly informative to explain volume or CSAmin, symbolic regression analyses were performed to determine the importance of variables to model the upper airway characteristics. Models converged quickly. Figure 3A,B presents the variables ranked by normalized fitness-weighted importance to explain the CSAmin and the volume, respectively. This metric is related to the importance of the variables, i.e., the proportion of equations in which these variables appear, weighted by their fit (the mean absolute error). All equations obtained for each experimental run were provided in Supplementary Tables S1 and S2. The distance PNS–LP mostly influenced both CSAmin and volume. Other outcomes were related to the hyoid bone (distance C3–H, angle H–Na–S), the soft palate (distance PNS–VSP), the tongue (distance BEP–A, BEP–TUV) or the mandibular or maxillary position (distance Na–B, angle S–Na–A). In order to evaluate the direction of the effect of these parameters on the volume or CSAmin, a sensitivity analysis was performed. Distances PNS–LP, BEP–TUV and angle H–Na–S had a negative mean magnitude on the CSAmin with −0.91, −0.14 and −0.07 (variables found in 10, 2 and 2 of the best models, respectively). Distance PNS–LP and angle H–Na–S had a negative mean magnitude on the airway volume with −0.97 and −0.39, respectively, meaning that an increase in the variable induces a decrease in the volume (variables found in 10/10 of the best models). Conversely, distances BEP–A, PNS–VSP and angle S–Na–A had a positive mean magnitude on the airway volume with 0.27, 0.29 and 0.37, respectively (variables found in 6 to 10 of the best models).

In summary, the results from symbolic regression analyses strengthen the results from the descriptive analysis. Soft tissues that are the most important to explain the upper airway are the horizontal soft palate, the tongue, and the position of the hyoid bone. The hard anatomical structure that is the most important to explain the upper airway is the anteroposterior position of the mandible and the maxilla related to the base of the skull.

## 4. Discussion

In this study, the aim was to assess the correlation between hard and soft anatomical parameters and their impact on the characteristics of the upper airway on CBCT scans.

The upper airway is a complex structure. The superior boundary of the upper airway is defined by a plane passing by PNS and ANS (to define the hard palate) and parallel to the FH plane [6,7,8,9,10,11,12,13,14,15,16,17,18]. In contrast, the position of the inferior boundary is not absolute. Indeed, some authors consider the BEP parallel to the FH plane [13,16,23], others consider it to be the superior part of the trachea [6,7,10,33], the anteroinferior point of C3 [34,35], the anteroinferior point of C2 [9,36,37] or the anteroinferior point of C4 [15,38]. In this study, the inferior boundary was defined as the anteroinferior point of C3 and parallel to the FH plane [34,35]. To compensate for the potential head incline during acquisition, all examinations were reoriented according to the FH and the midsagittal planes. To our knowledge, no study has performed an upper airway region analysis using validated soft and hard anatomical parameters simultaneously.

The upper airway is generally defined by its volume, its minimal section [7,8,9,15,16,32,36,37,39]. The volume of the upper airway and the CSAmin are obtained by automatic segmentation in Avizo software (Supplementary Text). In this study, the width of the nasal cavity was added as a hard-anatomical parameter even if such a parameter was not commonly reported in the literature. It made sense to add the nasopharynx, as its anatomy may participate directly or indirectly in the characteristics of the upper airway. All the other angles and distances were justified by the scientific literature [6,7,8,9,10,11,12,14,15,16,23]. The rationale for the mathematical computation of distances and angles from the spatial position of landmarks rather than measurements directly on Avizo was to improve the reproducibility of measures.

Several studies, such as that of de Oliveira et al., pointed out a more important reproducibility when using anatomical landmarks on a CBCT scan, if a protocol for operator training and calibration was followed [27,32]. Finally, the intra-observer reproducibility of this study was good to excellent with ICC values > 0.7. The possibility of both obtaining a 3D visualization from CBCT scans, and working on the three planes of space, may also have improved landmarks positioning more easily than using 2D radiography [1,17,19,20,21,22,25,40,41].

The literature reports that there are craniofacial morphological characteristics in OSA patients such as reduced upper airway space, anteroposterior position of the mandible (Na-B), abnormal long soft palate and tongue dimension, and low position of the hyoid bone [6,7,8,9,10,11,12,14,15,16,23,34,37,39,42,43,44]. Some authors have compared patients with and without OSA [7,9,10,11,16,23,32,36,37,39,43,44,45,46]. In this study, the analysis of the upper airway was carried out in a general population, irrespective of their medical pathology. However, CBCT exams were performed for medical reasons, such as sinus pathology and orthodontic disorders, and nothing excludes that the prescription of these examinations could not be directly or indirectly linked to an upper airway pathology (selection bias of the population).

The aim of the symbolic regression was to seek an optimal model between a set of different types of predefined mathematical functions and their combinations. This kind of methodology opens new perspectives in terms of flexibility and accuracy for statistical modeling [47]. Such an innovative approach has made it possible here to demonstrate the predominant importance of the horizontal soft palate to predict both the airway volume and minimal cross-section area. Our results are also in accordance with the literature. The soft tissues are predominately responsible for the reduction of the upper airway, especially the tongue, the soft palate and the hyoid bone position [8,9,16,32,44]. For the hard structures, the anteroposterior position of the mandible is also an important feature linked to the characteristics of the upper airway [8,12,34,35,37]. A significant correlation between the volume and the CSAmin, and its anteroposterior and lateral dimensions has been also reported [36].

This study shows that a few simple parameters, concerning both bone and soft tissues, can give information on the volume and restriction of the section of the airway. It is therefore important that the CBCT volume, often performed for dento-maxillary issues, be read in full with a systematic approach [48]. This implies strengthening the training of dental medical doctors to make them aware of incidental findings, and in particular of the airway and the risk of OSA. This will go together with the automation of linear or volume measurement by artificial intelligence [49,50]. Exposing the patient to the smallest possible amount of radiation is a major concern in medical imaging. If an acquisition is to be made specifically for the upper airway, CBCT low-dose protocols could be considered [51,52] as well as other types of imaging such as “Black Bone” magnetic resonance [53].

## 5. Conclusions

The upper airway is an important and complex anatomic structure in relationship with the development of pathogenesis such as OSA. In a general population, the soft tissues that are predominately responsible for the upper airway morphology are the soft palate and the hyoid bone position. For the hard structures, the anteroposterior of the mandible and the maxilla are also important features linked to the characteristics of the upper airway. Thus, although the volume of the airway is not accessible on all CBCT exams performed by dental practitioners, this study demonstrates that a small number of anatomical elements may be markers of the reduction of the upper airway, with potentially an increased risk of OSA. This could help the dentist refer the patient to a suitable physician.

## Figures and Tables

**Table 1:** Definitions of the anatomical landmarks.

Landmark Reference Lines	Definition (Anatomic Region)
A	Deepest anterior point in the concavity of the anterior maxilla
ANS	Anterior nasal spine, most anterior point of the nasal spine
B	Deepest anterior point in the concavity of the anterior mandible
Ba	Basion, most posteroinferior point on the clivus
BEP	Base of epiglottis, bottom of epiglottis crypt
C3	Most anterior point of the third cervical vertebral body
H	Most anterosuperior point of the hyoid bone
lCN	Left nasal cavity
lGo	Left mandibular gonion, angle of the left mandibular body
lOr	Left orbital, deepest point of the infraorbital margin: lateral-inferior contour of the left orbit
lTb	Left tuberosity, distal contour of the left maxillary tuberosity
MGNM	Foramen magnum, mid-posterior point of the large opening in the occipital bone
Me	Menton, most inferior point of the chin bone
Na	Nasion, anterior point at the frontonasal suture
Pg	Prognathion, most anterior point of the symphysis of the mandible
PNS	Posterior nasal spine, most posterior point of the nasal spine
rCN	Right nasal cavity
rGo	Right mandibular gonion, angle of the right mandibular body
rOr	Right orbital, deepest point of the infraorbital margin: lateral-inferior contour of the right orbit
rPo	Right porion, upper point of the right bony ear opening
rTb	Right tuberosity, distal contour of the right maxillary tuberosity
S	Sella, midpoint of the fossa hypophysealis
Tph	Pharyngeal hypertrophy
TUV	Tip of uvula, inferior point of caudal margin of the uvula at the mid-sagittal plane

**Table 2:** Definitions of the upper airway measurements.

Variable	Definition
Volume of the upper airway (Vol)	Superior boundary (i.e., the plane across PNS and ANS parallel to Frankfort plane (FH plane))—the inferior boundary (i.e., the plane across the anteroinferior point of the body of the 3rd cervical spinal vertebra parallel to the FH plane) in a mid-sagittal view
Minimum cross-sectional area (CSAmin)	The minimum cross-sectional area of the upper airway in an axial view
Lateral dimension of the CSAmin (Lat)	Width of CSAmin in a coronal view
Anteroposterior dimension of the CSAmin (Ap)	Length of CSAmin in a sagittal view

**Table 3:** Measurements computed from landmarks.

Variable	Definition
*Related to hard tissues*	
Mandible dimension	Mid-sagittal view, distance from: Me to rGo, in axial view distance between rGo and lGo
Anteroposterior position of maxilla and mandible	Mid-sagittal view, distance from Na to B, or from Na to A
Anteroposterior shift	Mid-sagittal view, distance from A to B projected on the Frankfort plane
Cranial basis dimension	Mid-sagittal view, distance from S to Na
Facial angle (SNPg)	Mid-sagittal view, angle formed by S-Na-Pg
FMA angle	Mid-sagittal view, angle formed by Frankfort plane and mandible plane (angle between rPo–rOr–Me–rGo)
Localization of hyoid bone	Mid-sagittal view, distance from C3 to H, Me to H, H to PNS, angle formed by H–S–Ba, H–Na–S
Maxilla dimension	Mid-sagittal view, distance between rTb and lTb
Nasal cavity dimension	Mid-sagittal view, distance from rCN to lCN or from Na to ANS
SNA angle	Mid-sagittal view, angle formed by S–Na–A
SNB angle	Mid-sagittal view, angle formed by S–Na–B
*Related to soft tissues*	
Dimension of the tongue	Mid-sagittal view, distance from BEP to A, from BEP to TUV
Horizontal soft palate (HSP)	Mid-sagittal view, distance from the PNS to the vertical line going through the most posterior contour of soft palate
Localization of soft palate	Mid-sagittal view, distance from PNS to TUV
Pharyngeal tonsils of soft tissue	Mid-sagittal view, distance from Ba to the most anterior point of pharyngeal hypertrophy (TPh)
Vertical soft palate (VSP)	Mid-sagittal view, distance from the horizontal line going through the PNS to the tip of soft palate

**Table 4:** Intraclass correlation coefficient (ICC) for the intra-observer reproducibility.

Variable	ICC Intra	*p*-Value
CSAmin	0.94 [0.85; 0.98]	<0.001
CSAmin width (Lat)	0.70 [0.37; 0.87]	<0.001
CSAmin length (Ap)	0.77 [0.49; 0.90]	<0.001
Volume	0.96 [0.91; 0.98]	<0.001
Anteroposterior position of maxilla (Na–A)	0.74 [0.42; 0.89]	<0.001
Anteroposterior position of mandible (Na–B)	0.92 [0.81; 0.97]	<0.001
Anteroposterior shift (A–B)	0.81 [0.58; 0.92]	<0.001
Cranial basis dimension (S–Na)	0.88 [0.71; 0.95]	<0.001
Dimension of tongue (height: BEP–A)	0.93 [0.78; 0.97]	<0.001
Dimension of tongue (width: BEP–TUV)	0.88 [0.72; 0.95]	<0.001
Facial angle (S–Na–Pg)	0.95 [0.87; 0.98]	<0.001
FMA angle	0.94 [0.81; 0.98]	<0.001
Horizontal soft palate (PNS–LP: HSP)	0.76 [0.47; 0.90]	<0.001
Length of the mandible (Me–rGo)	0.89 [0.74; 0.95]	<0.001
Localization of hyoid bone (C3–H)	0.97 [0.91; 0.99]	<0.001
Localization hyoid bone (H–Na–S angle)	0.96 [0.90; 0.98]	<0.001
Localization of hyoid bone (H–PNS)	0.98 [0.95; 0.99]	<0.001
Localization hyoid bone (H–S–Ba angle)	0.98 [0.94; 0.99]	<0.001
Localization of hyoid bone (Me–H)	0.91 [0.77; 0.96]	<0.001
Localization of hyoid bone (S–H)	0.98 [0.95; 0.99]	<0.001
Localization of soft palate (TUV–PNS)	0.73 [0.43; 0.89]	<0.001
Maxilla dimension (rTb–lTb)	0.83 [0.62; 0.93]	<0.001
Nasal dimension (width: rCN–lCN)	0.60 [0.23; 0.82]	<0.001
Nasal dimension (height: Na–ANS)	0.84 [0.60; 0.94]	<0.001
SNA angle	0.67 [0.34; 0.86]	<0.001
SNB angle	0.93 [0.84; 0.97]	<0.001
Thickness of soft tissue—pharyngeal hypertrophy (Ba-Tph)	0.74 [0.45; 0.89]	<0.001
Vertical soft palate (PNS–VSP)	0.50 [0.10; 0.77]	<0.001
Width of the mandible (rGo–lGo)	0.89 [0.33; 0.97]	<0.001

**Table 5:** Descriptive analysis of each variable using means, standard deviations, medians, and quartiles (N = 60).

Variable	Average ± SD	Median [Q1; Q3]	Min; Max
Age	39.9 ± 13.5	40 [27.5; 50.0]	22–72
Anteroposterior position of mandible (Na–B, mm)	94.6 ± 7.5	94.0 [90.5; 97.7]	77.1; 118
Anteroposterior position of maxilla (Na–A, mm)	57.1 ± 4.1	56.4 [54.2; 59.6]	47.3; 67.0
Anteroposterior shift (A–B, mm)	4.0 ± 4.5	4.5 [1.4; 6.9]	−12.1; 13.9
Cranial Basis dimension (S–Na, mm)	66.2 ± 4.1	64.8 [63.3; 68.5]	58.6; 77.9
CSAmin (mm${}^{2}$)	206 ± 123	190 [115; 275]	46; 618
CSAmin Lat (mm)	27.0 ± 7.0	26.0 [22.0; 32.7]	13.7; 44.6
CSAmin AP (mm)	10.2 ± 3.6	9.7 [7.7; 13.0]	3.1; 18.8
Dimension of tongue (height: BEP–A, mm)	85.7 ± 6.9	84.0 [80.3; 90.7]	75.0; 102
Dimension of tongue (width: BEP–TUV, mm)	30.0 ± 7.2	31.5 [25.1; 35.3]	15.5; 43.8
Facial Angle (S–Na–Pg, °)	79.0 ± 5.2	78.2 [75.9; 83.1]	68.7; 93.4
FMA Angle (°)	33.3 ± 6.5	33.2 [28.1; 37.7]	18.2; 48.1
Horizontal soft palate (PNS–LP: HSP, mm)	19.1 ± 4.5	19.0 [15.5; 21.8]	11.1; 34.9
Length of the mandible (Me–rGo, mm)	83.0 ± 6.4	83.2 [79.6; 87.2]	57.5; 95.8
Localization of hyoid bone (C3–H, mm)	34.6 ± 4.8	34.1 [31.1; 37.2]	25.8; 45.2
Localization of hyoid bone (H–S–Ba angle, °)	39.4 ± 6.4	39.3 [33.8; 43.6]	25.6; 54.7
Localization of hyoid bone (H–Na–S angle, °)	56.1 ± 4.3	56.0 [52.8; 59.2]	46.8; 68.6
Localization of hyoid bone (S–H, mm)	103 ± 8.8	102 [97.1; 109]	77.9; 122
Localization of hyoid bone (Me–H, mm)	42.0 ± 4.8	42.0 [38.9; 45.9]	27.7; 51.7
Localization of hyoid bone (H–PNS, mm)	61.3 ± 6.9	61.4 [56.3; 66.0]	44.2; 76.0
Localization of soft palate (TUV–PNS, mm)	36.5 ± 4.3	36.9 [33.3; 39.4]	27.1; 44.9
Maxilla dimension (rTb–lTb, mm)	49.1 ± 4.0	49.5 [45.9; 51.7]	41.7; 58.3
Nasal dimension (height: Na–ANS, mm)	50.3 ± 3.6	49.9 [48.0; 52.5]	42.2; 60.5
Nasal dimension (width: rCN–lCN, mm)	20.5 ± 5.6	19.8 [17.5; 22.0]	12.9; 54.4
SNA Angle (°)	81.8 ± 4.4	81.6 [78.5; 84.5]	72.8; 92.2
SNB Angle (°)	77.8 ± 5.0	77.3 [74.7; 81.4]	66.6; 96.5
Thickness of soft tissue—pharyngeal hypertrophy (Ba–Tph, mm)	18.4 ± 5.1	17.5 [14.9; 20.7]	11.3; 38.9
Vertical soft palate (PNS–VSP, mm)	36.2 ± 5.0	35.5 [33.2; 38.6]	26.6; 49.0
Volume (mm${}^{3}$)	14,460 ± 7399	13,645 [8495; 18,092]	1614; 40,720
Width of the mandible (lGo–rGo, mm)	92.0 ± 5.8	92.1 [88.4; 96.3]	78.8; 107

## Data Availability

The data that support the findings of this study are available from the corresponding author upon reasonable request.

## Supplementary Materials

The following supporting information can be downloaded at: https://www.mdpi.com/article/10.3390/jcm12010084/s1.

## References

[1] Yitschaky O., Redlich M., Abed Y., Faerman M., Casap N., Hiller N. (2011). Comparison of common hard tissue cephalometric measurements between computed tomography 3D reconstruction and conventional 2D cephalometric images. Angle Orthod..

[2] Bruwier A., Poirrier R., Albert A., Maes N., Limme M., Charavet C., Milicevic M., Raskin S., Poirrier A.L. (2016). Analyse tridimensionnelle des os craniofaciaux et des tissus mous dans l’apnée obstructive du sommeil utilisant la tomographie volumétrique à faisceau conique. Int. Orthod..

[3] Friedlander-Barenboim S., Hamed W., Zini A., Yarom N., Abramovitz I., Chweidan H., Finkelstein T., Almoznino G. (2021). Patterns of Cone-Beam Computed Tomography (CBCT) Utilization by Various Dental Specialties: A 4-Year Retrospective Analysis from a Dental and Maxillofacial Specialty Center. Healthcare.

[4] Maret D., Vergnes J.N., Peters O.A., Peters C., Nasr K., Monsarrat P. (2020). Recent Advances in Cone-beam CT in Oral Medicine. Curr. Med. Imaging.

[5] Portelli M., Militi A., Lo Giudice A., Lo Giudice R., Fastuca R., Ielo I., Mongelli V., Lo Giudice G., Martintoni A., Manuelli M. (2018). Standard and low-dose cone beam computer tomography protocol for orthognatodontic diagnosis: A comparative evaluation. J. Biol. Regul. Homeost. Agents.

[6] Zimmerman J.N., Lee J., Pliska B.T. (2017). Reliability of upper pharyngeal airway assessment using dental CBCT: A systematic review. Eur. J. Orthod..

[7] Buchanan A., Cohen R., Looney S., Kalathingal S., De Rossi S. (2016). Cone-beam CT analysis of patients with obstructive sleep apnea compared to normal controls. Imaging Sci. Dent..

[8] Guijarro-Martínez R., Swennen G.R.J. (2011). Cone-beam computerized tomography imaging and analysis of the upper airway: A systematic review of the literature. Int. J. Oral Maxillofac. Surg..

[9] Enciso R., Nguyen M., Shigeta Y., Ogawa T., Clark G.T. (2010). Comparison of cone-beam CT parameters and sleep questionnaires in sleep apnea patients and control subjects. Oral Surg. Oral Med. Oral Pathol. Oral Radiol. Endod..

[10] Alsufyani N.A., Noga M.L., Witmans M., Major P.W. (2017). Upper airway imaging in sleep-disordered breathing: Role of cone-beam computed tomography. Oral Radiol..

[11] Alsufyani N.A., Al-Saleh M.A.Q., Major P.W. (2013). CBCT assessment of upper airway changes and treatment outcomes of obstructive sleep apnoea: A systematic review. Sleep Breath..

[12] Glupker L., Kula K., Parks E., Babler W., Stewart K., Ghoneima A. (2015). Three-dimensional computed tomography analysis of airway volume changes between open and closed jaw positions. Am. J. Orthod. Dentofac. Orthop..

[13] Chen H., Aarab G., Parsa A., de Lange J., van der Stelt P.F., Lobbezoo F. (2016). Reliability of three-dimensional measurements of the upper airway on cone beam computed tomography images. Oral Surg. Oral Med. Oral Pathol. Oral Radiol..

[14] Jiang Y.Y. (2016). Correlation between hyoid bone position and airway dimensions in Chinese adolescents by cone beam computed tomography analysis. Int. J. Oral Maxillofac. Surg..

[15] Alsufyani N.A., Flores-Mir C., Major P.W. (2012). Three-dimensional segmentation of the upper airway using cone beam CT: A systematic review. Dentomaxillofac. Radiol..

[16] Chen H., Li Y., Reiber J.H., de Lange J., Tu S., van der Stelt P., Lobbezoo F., Aarab G. (2018). Analyses of aerodynamic characteristics of the oropharynx applying CBCT: Obstructive sleep apnea patients versus control subjects. Dentomaxillofac. Radiol..

[17] Eslami E., Katz E.S., Baghdady M., Abramovitch K., Masoud M.I. (2017). Are three-dimensional airway evaluations obtained through computed and cone-beam computed tomography scans predictable from lateral cephalograms? A systematic review of evidence. Angle Orthod..

[18] Ghoneima A., Kula K. (2013). Accuracy and reliability of cone-beam computed tomography for airway volume analysis. Eur. J. Orthod..

[19] Schlicher W., Nielsen I., Huang J.C., Maki K., Hatcher D.C., Miller A.J. (2012). Consistency and precision of landmark identification in three-dimensional cone beam computed tomography scans. Eur. J. Orthod..

[20] Lagravère M.O., Low C., Flores-Mir C., Chung R., Carey J.P., Heo G., Major P.W. (2010). Intraexaminer and interexaminer reliabilities of landmark identification on digitized lateral cephalograms and formatted 3-dimensional cone-beam computerized tomography images. Am. J. Orthod. Dentofac. Orthop..

[21] Souza K.R.S.d., Oltramari-Navarro P.V.P., Navarro R.d.L., Conti A.C.d.C.F., Almeida M.R.d. (2013). Reliability of a method to conduct upper airway analysis in cone-beam computed tomography. Braz. Oral Res..

[22] Nalçaci R., Oztürk F., Sökücü O. (2010). A comparison of two-dimensional radiography and three-dimensional computed tomography in angular cephalometric measurements. Dentomaxillofac. Radiol..

[23] Chen H., Aarab G., de Ruiter M.H.T., de Lange J., Lobbezoo F., van der Stelt P.F. (2016). Three-dimensional imaging of the upper airway anatomy in obstructive sleep apnea: A systematic review. Sleep Med..

[24] Osorio F., Perilla M., Doyle D.J., Palomo J.M. (2008). Cone beam computed tomography: An innovative tool for airway assessment. Anesth. Analg..

[25] Ludlow J.B., Gubler M., Cevidanes L., Mol A. (2009). Precision of cephalometric landmark identification: Cone-beam computed tomography vs conventional cephalometric views. Am. J. Orthod. Dentofac. Orthop..

[26] Naji P., Alsufyani N.A., Lagravère M.O. (2014). Reliability of anatomic structures as landmarks in three-dimensional cephalometric analysis using CBCT. Angle Orthod..

[27] de Oliveira A.E.F., Cevidanes L.H.S., Phillips C., Motta A., Burke B., Tyndall D. (2009). Observer reliability of three-dimensional cephalometric landmark identification on cone-beam computerized tomography. Oral Surg. Oral Med. Oral Pathol. Oral Radiol. Endod..

[28] Cheng E., Chen J., Yang J., Deng H., Wu Y., Megalooikonomou V., Gable B., Ling H. Automatic Dent-landmark detection in 3-D CBCT dental volumes. Proceedings of the 2011 Annual International Conference of the IEEE Engineering in Medicine and Biology Society.

[29] Alsufyani N.A., Dietrich N.H., Lagravère M.O., Carey J.P., Major P.W. (2014). Cone beam computed tomography registration for 3-D airway analysis based on anatomic landmarks. Oral Surg. Oral Med. Oral Pathol. Oral Radiol..

[30] Bobak C.A., Barr P.J., O’Malley A.J. (2018). Estimation of an inter-rater intra-class correlation coefficient that overcomes common assumption violations in the assessment of health measurement scales. BMC Med. Res. Methodol..

[31] Vladislavleva K., Veeramachaneni K., Burland M., Parcon J., O’Reilly U.M. (2010). Knowledge mining with genetic programming methods for variable selection in flavor design. Proceedings of the 12th Annual Conference on Genetic and Evolutionary Computation—GECCO’10.

[32] Neelapu B.C., Kharbanda O.P., Sardana H.K., Balachandran R., Sardana V., Kapoor P., Gupta A., Vasamsetti S. (2017). Craniofacial and upper airway morphology in adult obstructive sleep apnea patients: A systematic review and meta-analysis of cephalometric studies. Sleep Med. Rev..

[33] Alves M., Baratieri C., Mattos C.T., Brunetto D., Fontes R.d.C., Santos J.R.L., Ruellas A.C.d.O. (2012). Is the airway volume being correctly analyzed?. Am. J. Orthod. Dentofac. Orthop..

[34] Grauer D., Cevidanes L.S.H., Styner M.A., Ackerman J.L., Proffit W.R. (2009). Pharyngeal airway volume and shape from cone-beam computed tomography: Relationship to facial morphology. Am. J. Orthod. Dentofac. Orthop..

[35] Li L., Wu W., Yan G., Liu L., Liu H., Li G., Li J., Liu D. (2016). Analogue simulation of pharyngeal airflow response to Twin Block treatment in growing patients with Class II1 and mandibular retrognathia. Sci. Rep..

[36] Ogawa T., Enciso R., Shintaku W.H., Clark G.T. (2007). Evaluation of cross-section airway configuration of obstructive sleep apnea. Oral Surg. Oral Med. Oral Pathol. Oral Radiol. Endod..

[37] El H., Palomo J.M. (2010). Measuring the airway in 3 dimensions: A reliability and accuracy study. Am. J. Orthod. Dentofac. Orthop..

[38] Shah D.H., Kim K.B., McQuilling M.W., Movahed R., Shah A.H., Kim Y.I. (2016). Computational fluid dynamics for the assessment of upper airway changes in skeletal Class III patients treated with mandibular setback surgery. Angle Orthod..

[39] Indriksone I., Jakobsone G. (2014). The upper airway dimensions in different sagittal craniofacial patterns: A systematic review. Stomatologija.

[40] Lisboa C.d.O., Masterson D., da Motta A.F.J., Motta A.T. (2015). Reliability and reproducibility of three-dimensional cephalometric landmarks using CBCT: A systematic review. J. Appl. Oral Sci..

[41] Jung P.K., Lee G.C., Moon C.H. (2015). Comparison of cone-beam computed tomography cephalometric measurements using a midsagittal projection and conventional two-dimensional cephalometric measurements. Korean J. Orthod..

[42] Hatcher D.C. (2012). Cone beam computed tomography: Craniofacial and airway analysis. Dent. Clin. N. Am..

[43] Kikuchi M., Higurashi N., Miyazaki S., Itasaka Y. (2000). Facial patterns of obstructive sleep apnea patients using Ricketts’ method. Psychiatry Clin. Neurosci..

[44] Schwab R.J., Pasirstein M., Pierson R., Mackley A., Hachadoorian R., Arens R., Maislin G., Pack A.I. (2003). Identification of upper airway anatomic risk factors for obstructive sleep apnea with volumetric magnetic resonance imaging. Am. J. Respir. Crit. Care Med..

[45] deBerry Borowiecki B., Kukwa A., Blanks R.H. (1988). Cephalometric analysis for diagnosis and treatment of obstructive sleep apnea. Laryngoscope.

[46] Noud M., Hovis K., Gelbard A., Sathe N.A., Penson D.F., Feurer I.D., McPheeters M.L., Francis D.O. (2017). Patient-reported outcome measures in upper airway–related dyspnea. JAMA Otolaryngol. Head Neck Surg..

[47] Hassoumi A., Peysakhovich V., Hurter C. (2019). Improving eye-tracking calibration accuracy using symbolic regression. PLoS ONE.

[48] Miracle A., Mukherji S. (2009). Conebeam CT of the Head and Neck, Part 2: Clinical Applications. Am. J. Neuroradiol..

[49] Alsubai S. (2022). A Critical Review on the 3D Cephalometric Analysis Using Machine Learning. Computers.

[50] Orhan K., Shamshiev M., Ezhov M., Plaksin A., Kurbanova A., Ünsal G., Gusarev M., Golitsyna M., Aksoy S., Mısırlı M. (2022). AI-based automatic segmentation of craniomaxillofacial anatomy from CBCT scans for automatic detection of pharyngeal airway evaluations in OSA patients. Sci. Rep..

[51] Leonardi R., Giudice A.L., Farronato M., Ronsivalle V., Allegrini S., Musumeci G., Spampinato C. (2021). Fully automatic segmentation of sinonasal cavity and pharyngeal airway based on convolutional neural networks. Am. J. Orthod. Dentofac. Orthop..

[52] Feragalli B., Rampado O., Abate C., Macrì M., Festa F., Stromei F., Caputi S., Guglielmi G. (2017). Cone beam computed tomography for dental and maxillofacial imaging: Technique improvement and low-dose protocols. Radiol. Med..

[53] Dremmen M., Wagner M., Bosemani T., Tekes A., Agostino D., Day E., Soares B., Huisman T. (2017). Does the Addition of a “Black Bone” Sequence to a Fast Multisequence Trauma MR Protocol Allow MRI to Replace CT after Traumatic Brain Injury in Children?. Am. J. Neuroradiol..

