# Supplementary material for: Machine Learning Analysis of the Anatomical Parameters of the Upper Airway Morphology: A Retrospective Study from Cone-Beam CT Examinations in a French Population
Source: J Clin Med. 2022 Dec 22;12(1):84. doi: 10.3390/jcm12010084 (PMC9820916; doi:10.3390/jcm12010084)
Supplement: Supplementary file 1 [file jcm-12-00084-s001.zip › jcm-2060100-supplementary.pdf]

Supplementary Text

Supplementary Text S1: Protocol under Avizo 8.1.

1. Orientation

The anonymized scan data were opened using Avizo 8.1 and visualized using the orthoslice tool (values between -500 and 2000), and the isosurface tool (threshold from 500 to 600). First, the scans were re-oriented according to the Frankfort plane, defined by three anatomic landmarks (rPo, rOr and lOr).

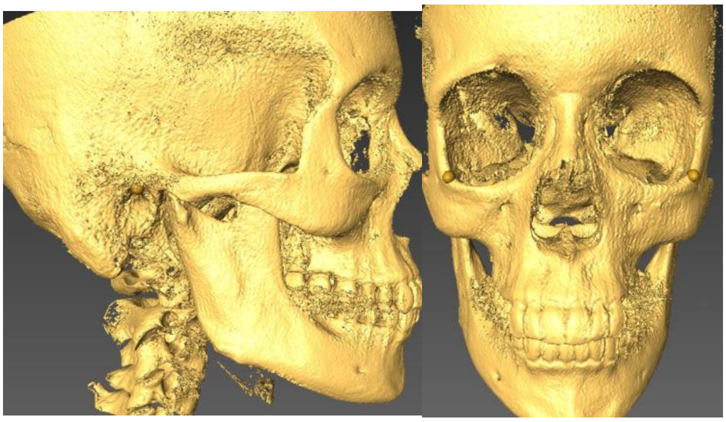

Position of three anatomic landmarks on the isosurface (rPo, rOr, lOr).

Second, the scans were re-oriented according to the midsagittal plane, defined by three anatomic landmarks (Na, ANS and MGNM).

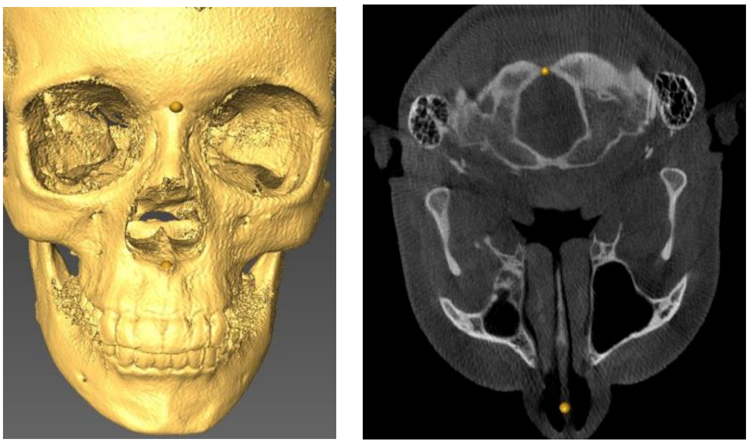

Position of 3 landmarks on the isosurface and orthoslice (Na, ANS, FM).

2. Volume Segmentation

The segmentation was performed semi-automatically using the software tool “Image Segmentation - Edit New label field”. The threshold tool was adjusted to around 440, and the upper and lower boundaries of the upper airway were placed on the midsagittal orthoslice. Then, the upper airway was selected on the sagittal view. The cross-sectional areas were computed using the tool “measure and analysis” and the minimal value was considered (CSAmin). The corresponding slice of the airway was used to compute the width and the length.

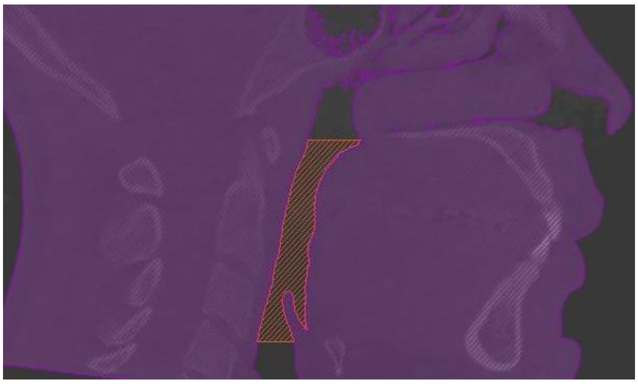

Volume segmentation of the upper airway in the sagittal plane.

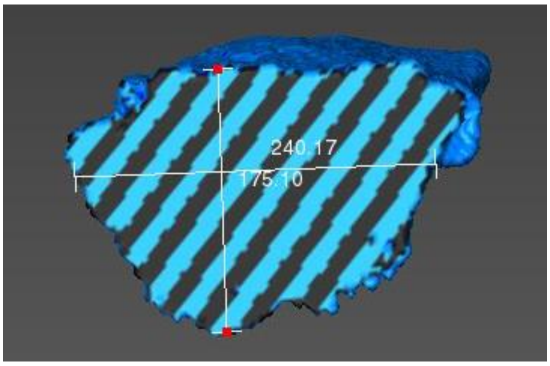

Length (anteroposterior) and width (lateral) of the CSAmin

3. Anatomical landmarks

Using the software tool “Create object – Landmarks”, the observer placed all the anatomical landmarks in the same order (from 1 to 24) using the three orthoslices (midsagittal, axial and frontal views) and the isosurface.

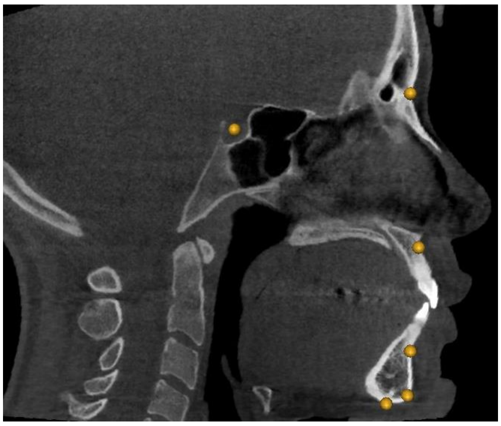

Na, Pg, S, A, B and Me

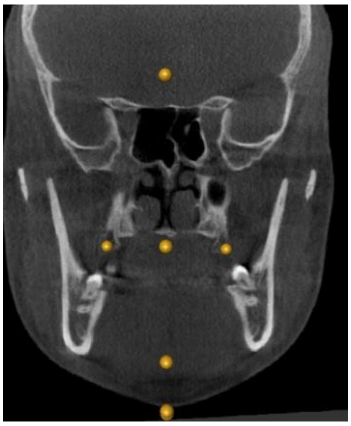

rTb and lTb

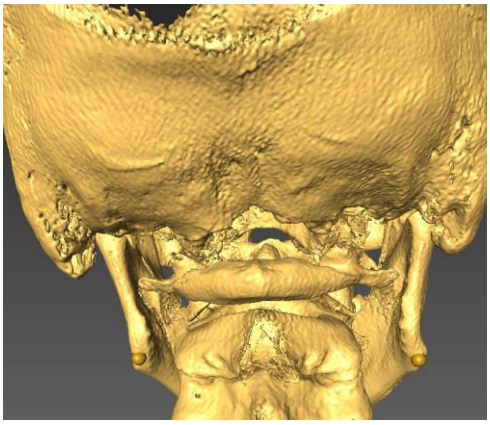

rGo and lGo

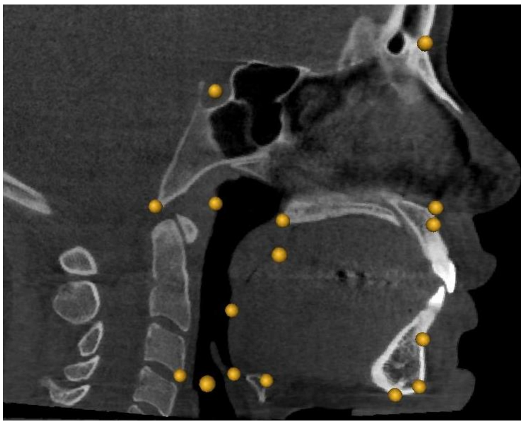

PNS, ANS, BEP, TUV, Ba, Tph, H and C3

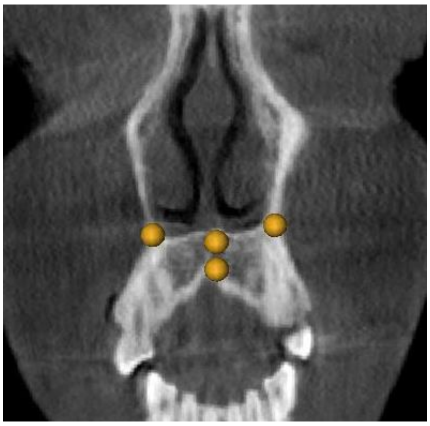

rCN, lCN

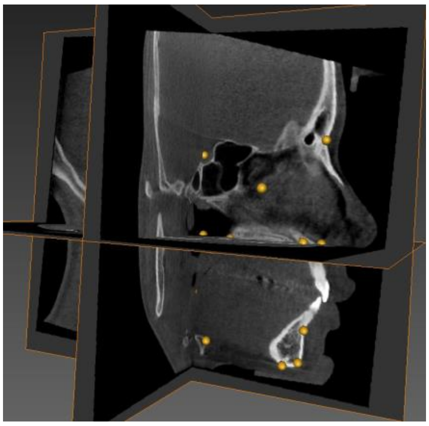

LP at the intersection of the three planes: midsagittal plane; axial plane passing through the PNS-ANS; frontal plane on the most posterior part of the soft palate

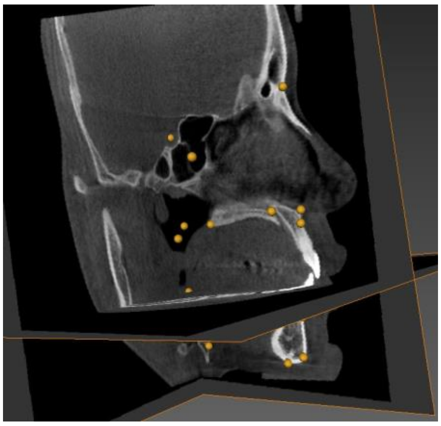

VSP at the intersection of the three planes: midsagittal plane; axial plane passing through the TUV; frontal plane on the most posterior part of the soft palate

Positioning of some landmarks.

Supplementary Text S2: Normalized fitness-weighted variable importance and magnitude of effects

Definition of the normalized fitness-weighted variable importance

Fitness-weighted variable importance FWI is calculated using all models. It first uniformly distributes the fitness (mean absolute error) of each model $M = \{j = 1, \ldots, m\}$ over all d variables present in it ($\delta(ki, Mj)$). Then, it sums up the scores over all models.

$$FWI\ (xi, M) = \sum_{j=1}^{m} \frac{fitness(Mj)}{\sum_{i=1}^{d} \delta(ki, Mj)} \delta(ki, Mj)$$

The normalized fitness-weighted variable importance NFWI was used.

$$NFWI\ (xi, M) = \frac{FWI\ (xi, M)}{\sum_i FWI(ki, M)} .100\%$$

Definition of the magnitude of effects

For a model equation of the form $z = f(x, y \ldots)$, the positive magnitude is defined as $\overline{\left|\frac{\partial z}{\partial x}\right|} . \frac{\sigma x}{\sigma z}$, at all points where $\frac{\partial z}{\partial x} > 0$ and the negative magnitude as $\overline{\left|\frac{\partial z}{\partial x}\right|} . \frac{\sigma x}{\sigma z}$, at all points where $\frac{\partial z}{\partial x} < 0$.

$\frac{\partial z}{\partial x}$ is the partial derivative of z with respect to x,

σx is the standard deviation of x in the input data,

σz is the standard deviation of z

The mean magnitude of effects for each contributing variable was computed from the best model equation of each experiment. Magnitude of effects means that when the variable increases, there is an increase (positive magnitude) or a decrease (negative magnitude) in the target variable.

Supplementary Table S1: Results for the symbolic regression analysis of CSAmin. Metrics associated for each solution found for each of the 10 experiments in Eureqa software.

Experiment 1

R^2 Goodness of Fit	Correlation Coefficient	Maximum Error	Mean Squared Error	Mean Absolute Error	Complexity	Fit	Solution
0.55	0.80	100	3377	46	15	0.57	CSA = 206 + 122*(0.025 + 0.0856*((distanceNa-ANS) - 50.3) - 0.1111*((distancePNS-LP) - 19.07) - 0.0162*((distanceNa-ANS) - 50.32)*((angleS-Na-A) - 81.77))
0.56	0.80	105	3302	47	13	0.58	CSA = 206 + 122*(0.0706*((distanceNa-ANS) - 50) - 0.11*((distancePNS-LP) - 19) - 0.0162*((distanceNa-ANS) - 50)*((angleS-Na-A) - 81))
0.44	0.67	121	4273	54	7	0.67	CSA = 206 + 122*(0.11*((distanceNa-ANS) - 50.3) - 0.132*((distancePNS-LP) - 19.1))
0.36	0.65	174	4869	55	3	0.68	CSA = 206 - 12.97*((distancePNS-LP) - 19.07)

Experiment 2

R^2 Goodness of Fit	Correlation Coefficient	Maximum Error	Mean Squared Error	Mean Absolute Error	Complexity	Fit	Solution
0.56	0.78	104	3315	50	7	0.62	CSA = 206 + 122*(0.0489*((anglerOr-rPo-Me-rGo) - 33.32) - 0.11*((distancePNS-LP) - 19.07))
0.36	0.65	175	4841	54	3	0.67	CSA = 206 - 13.37*((distancePNS-LP) - 19.07)

Experiment 3

R^2 Goodness of Fit	Correlation Coefficient	Maximum Error	Mean Squared Error	Mean Absolute Error	Complexity	Fit	Solution
0.34	0.62	136	5023	56	11	0.69	CSA = 62 + 10.99*(distanceC3-H) - 0.237*(distancePNS-LP)*(angleH-Na-S)
0.47	0.74	119	3976	56	8	0.69	CSA = (distanceNa-ANS)*(angleS-Na-A)/(distancePNS-LP) - Age
0.41	0.64	161	4449	56	7	0.69	CSA = 471 - 0.257*(distancePNS-LP)*(angleH-Na-S)
0.35	0.65	167	4957	58	5	0.72	CSA = 422 - 11.42*(distancePNS-LP)
0.30	0.63	155	5272	65	4	0.80	CSA = 3625/(distancePNS-LP)
-0.03	0.01	144	7822	78	3	0.97	CSA = 5.31*(distanceC3-H)

Experiment 4

R^2 Goodness of Fit	Correlation Coefficient	Maximum Error	Mean Squared Error	Mean Absolute Error	Complexity	Fit	Solution
0.40	0.65	166	4569	54	5	0.68	CSA = 446 - 13.03*(distancePNS-LP)
0.20	0.63	178	6047	64	4	0.79	CSA = 3985 /(distancePNS-LP)
-0.02	0.14	150	7745	78	3	0.97	CSA = 2.09*(distanceNa-B)

Experiment 5

R^2 Goodness of Fit	Correlation Coefficient	Maximum Error	Mean Squared Error	Mean Absolute Error	Complexity	Fit	Solution
0.48	0.72	131	3953	55	7	0.68	CSA = 347 - 0.25*(distanceBEP-TUV)*(distancePNS-LP)
0.38	0.65	159	4694	58	5	0.72	CSA = 412 - 11.27*(distancePNS-LP)
0.26	0.63	168	5618	64	4	0.79	CSA = 3828/(distancePNS-LP)
-0.04	0.01	148	7878	79	3	0.98	CSA = 5.48*(distanceC3-H)

Experiment 6

R^2 Goodness of Fit	Correlation Coefficient	Maximum Error	Mean Squared Error	Mean Absolute Error	Complexity	Fit	Solution
0.41	0.64	167	4485	55	7	0.68	CSA = 484 - 0.27*(distancePNS-LP)*(angleH-Na-S)
0.38	0.65	162	4706	57	5	0.71	CSA = 420 - 11.64*(distancePNS-LP)
0.30	0.63	157	5320	64	4	0.80	CSA = 3664 /(distancePNS-LP)
-0.04	0.01	148	7875	79	3	0.98	CSA = 5.47*(distanceC3-H)

Experiment 7

R^2 Goodness of Fit	Correlation Coefficient	Maximum Error	Mean Squared Error	Mean Absolute Error	Complexity	Fit	Solution
0.54	0.83	107	3494	51	11	0.64	CSA = 7.58*(distanceNa-ANS) - 41 - 0.26*(distanceBEP-TUV)*(distancePNS-LP)
0.47	0.74	119	3976	56	8	0.69	CSA = (distanceNa-ANS)*(angleS-Na-A)/(distancePNS-LP) - Age
0.46	0.72	116	4053	56	7	0.70	CSA = 325 - 0.237*(distanceBEP-TUV)*(distancePNS-LP)
0.32	0.64	178	5121	57	6	0.71	CSA = 4513/(distancePNS-LP) - Age

0.39	0.65	158	4618	57	5	0.71	CSA = 416 - 11.6*(distancePNS-LP)
0.30	0.63	155	5274	65	4	0.80	CSA = 3627/(distancePNS-LP)
0.00	0.44	150	7603	77	3	0.95	CSA = 2.41*(angleS-Na-A)

Experiment 8

R^2 Goodness of Fit	Correlation Coefficient	Maximum Error	Mean Squared Error	Mean Absolute Error	Complexity	Fit	Solution
0.43	0.66	124	4286	53	11	0.66	CSA = (distanceNa-ANS)*sqrt((distanceNa-B)) - 15.1*(distancePNS-LP)
0.38	0.64	162	4675	54	7	0.67	CSA = 4.85*(distanceNa-B) - 13.1*(distancePNS-LP)
0.36	0.65	171	4808	55	5	0.68	CSA = 447 - 12.75*(distancePNS-LP)
-0.02	0.18	157	7750	75	3	0.93	CSA = 4.67*(angleH-S-Ba)

Experiment 9

R^2 Goodness of Fit	Correlation Coefficient	Maximum Error	Mean Squared Error	Mean Absolute Error	Complexity	Fit	Solution
0.40	0.65	170	4558	52	5	0.65	CSA = 467 - 14.1*(distancePNS-LP)
0.28	0.63	161	5421	64	4	0.80	CSA = 3729 /(distancePNS-LP)
-0.02	0.14	149	7743	78	3	0.97	CSA = 2.09*(distanceNa-B)

Experiment 10

R^2 Goodness of Fit	Correlation Coefficient	Maximum Error	Mean Squared Error	Mean Absolute Error	Complexity	Fit	Solution
0.34	0.65	179	5020	55	5	0.68	CSA = 467 - 13.5*(distancePNS-LP)
0.30	0.63	155	5277	65	4	0.80	CSA = 3629 /(distancePNS-LP)
-0.02	0.14	149	7737	78	3	0.97	CSA = 2.08*(distanceNa-B)

Supplementary Table S2: Results for the symbolic regression analysis of Volume. Metrics associated for each solution found for each of the 10 experiments in Eureqa.

Experiment 1

R^2 Goodness of Fit	Correlation Coefficient	Maximum Error	Mean Squared Error	Mean Absolute Error	Complexity	Fit	Solution
0.67	0.85	4967	3932065	1335	27	0.48	VOL = 14461 + 7336*(0.0527*((angleS-Na-A) - 81.77) + 0.0483*((distancePNS-VSP) - 36.18) + 0.0466*(distanceBEPA - 85.71) - 0.017*((distanceS-Na) - 66.2) - 0.0187*((distancerCN-lCN) - 20.52) - 0.0397*((angleH-Na-S) - 56.11) - 0.126*((distancePNS-LP) - 19.07))
0.55	0.75	6050	5358400	1609	21	0.57	VOL = 14462 + 7336*(0.0421 + 0.0389*(distanceBEPA - 85.71) + 0.0322*((distancePNS-VSP) - 36.18) - 0.0305*((distanceS-Na) - 66.2) - 0.0543*((angleH-Na-S) - 56.11) - 0.146*((distancePNS-LP) - 19.07))
0.52	0.73	5365	5676715	1848	19	0.66	VOL = 14462 + 7336*(0.0625*(distanceBEPA - 85.71) + 0.056*((distancePNS-VSP) - 36.18) - 0.0549*((angleH-Na-S) - 56.11) - 0.071*((distanceS-Na) - 66.2) - 0.13*((distancePNS-LP) - 19.1))
0.26	0.66	8313	8817206	1999	17	0.71	VOL = 14462 + 7336*(0.0279 + 0.0648*(distanceBEPA - 85.71) - 0.0363*((distanceS-Na) - 66.2) - 0.0791*((angleH-Na-S) - 56.11) - 0.181*((distancePNS-LP) - 19.07))
0.58	0.85	4255	5027672	2011	15	0.72	VOL = 14462 + 7336*(0.0705*((angleS-Na-A) - 81.8) + 0.0553*((distancePNS-VSP) - 36.18) - 0.064*((angleH-Na-S) - 56.11) - 0.118*((distancePNS-LP) - 19.07))
0.29	0.62	7137	8439873	2046	5	0.73	VOL = 14462 + 7336*(-0.118 - 0.101*((distancePNS-LP) - 19.07))
0.12	0.62	7973	10406930	2428	3	0.87	VOL = 14462 - 732*((distancePNS-LP) - 19.0728775932203)

Experiment 2

R^2 Goodness of Fit	Correlation Coefficient	Maximum Error	Mean Squared Error	Mean Absolute Error	Complexity	Fit	Solution
0.68	0.84	3510	3803659	1576	21	0.56	VOL = 14462 + 7336*(0.0706*((angleS-Na-A) - 81.77) + 0.049*(distanceBEPA - 85.71) + 0.0235*((distanceBa-Tph) - 18.36) - 0.225 - 0.0899*((angleH-Na-S) - 56.11) - 0.142*((distancePNS-LP) - 19.07))
0.63	0.83	4028	4403061	1619	19	0.58	VOL = 14461 + 7336*(0.0737*((distancePNS-VSP) - 36.18) + 0.0487*((distanceMe-H) - 41.96) - 0.062*((angleH-Na-S) - 56.11) - 0.0773*((distanceS-Na) - 66.2) - 0.127*((distancePNS-LP) - 19.07))
0.50	0.73	5419	5984826	1986	15	0.71	VOL = 14462 + 7336*(0.058*(distanceBEPA - 85.71) + 0.0438*((distancePNS-VSP) - 36.2) - 0.0539*((distanceS-Na) - 66.2) - 0.141*((distancePNS-LP) - 19.1))
0.29	0.63	7629	8450677	2081	9	0.74	VOL = 14462 + 7336*(0.038*(distanceBEPA - 85.71) - 0.0787 - 0.14*((distancePNS-LP) - 19.1))

0.25	0.62	7380	8834290	2145	5	0.77	VOL = 14462 + 7336*(-0.0974 - 0.105*((distancePNS-LP) - 19.1))
0.12	0.62	8001	10439190	2439	3	0.87	VOL = 14462 - 741*((distancePNS-LP) - 19.1)

Experiment 3

R^2 Goodness of Fit	Correlation Coefficient	Maximum Error	Mean Squared Error	Mean Absolute Error	Complexity	Fit	Solution
0.65	0.84	5103	4174416	1243	11	0.44	VOL = 4.84*distanceBEPA*(angleS-Na-A) - 19.2*(distancePNS-LP)*(angleH-Na-S)
0.45	0.78	6167	6549372	2047	9	0.73	VOL = 6.32*(distanceNa-ANS)*(angleS-Na-A) - 644*(distancePNS-LP)
0.27	0.62	7141	8631121	2083	5	0.74	VOL = 26395 - 656*(distancePNS-LP)
0.26	0.57	7043	8812454	2102	4	0.75	VOL = 251783/(distancePNS-LP)
0.20	0.69	6350	9526165	2355	3	0.84	VOL = 164*(angleS-Na-B)

Experiment 4

R^2 Goodness of Fit	Correlation Coefficient	Maximum Error	Mean Squared Error	Mean Absolute Error	Complexity	Fit	Solution
0.77	0.89	3564	2671211	1144	17	0.41	VOL = 5591+ 0.117*distanceBEPA*(distancePNS-VSP)*(angleS-Na-A) - 0.549*(distancePNS-LP)*(distancePNS-VSP)*(angleH-Na-S)
0.69	0.84	4721	3694196	1254	11	0.45	VOL = 4.47*distanceBEPA*(angleS-Na-A) - 16.93*(distancePNS-LP)*(angleH-Na-S)
0.40	0.64	5572	7065973	1947	9	0.70	VOL = 15762 + 356*(distancePNS-VSP) - 807*(distancePNS-LP)
0.29	0.57	6735	8433744	2029	4	0.72	VOL = 246920/(distancePNS-LP)

Experiment 5

R^2 Goodness of Fit	Correlation Coefficient	Maximum Error	Mean Squared Error	Mean Absolute Error	Complexity	Fit	Solution
0.71	0.86	4330	3459661	1151	15	0.41	VOL = distanceBEPA*(angleS-Na-A)*log((angleS-Na-A)) - 16.6*(distancePNS-LP)*(angleH-Na-S)
0.66	0.84	4907	3983724	1267	11	0.45	VOL = 4.78*distanceBEPA*(angleS-Na-A) - 18.9*(distancePNS-LP)*(angleH-Na-S)
0.50	0.79	5991	5902769	1929	9	0.69	VOL = 6.64*(distanceNa-ANS)*(angleS-Na-A) - 738*(distancePNS-LP)
0.27	0.57	6939	8669793	2072	4	0.74	VOL = 250142/(distancePNS-LP)
0.11	0.75	6740	10537413	2367	3	0.85	VOL = 164*(angleS-Na-A)

Experiment 6

R^2 Goodness of Fit	Correlation Coefficient	Maximum Error	Mean Squared Error	Mean Absolute Error	Complexity	Fit	Solution
0.69	0.86	4673	3673672	1371	30	0.49	VOL = 43.4*(distancePNS-LP)*(distancePNS-VSP) + 7.56*(distanceNa-ANS)*(angleS-Na-A) + 110/((distanceBEP-TUV) - 34.48) - 25850 - 0.0281*(distancePNS-VSP)*(angleH-Na-S)*(distancePNS-LP)^2
0.63	0.80	4421	4348239	1531	19	0.55	VOL = 397*(distancePNS-VSP) + 9.75*(distanceNa-ANS)*(angleS-Na-A) - 691*(distancePNS-LP) - 9.75*(distanceNa-ANS)*(angleH-Na-S)
0.64	0.82	5026	4309244	1537	15	0.55	VOL = 13336 + 8.73*(distanceNa-ANS)*(angleS-Na-A) - 395*(angleH-Na-S) - 722*(distancePNS-LP)
0.41	0.77	6977	7045535	1859	13	0.66	VOL = 11.4*(distanceNa-ANS)*(angleS-Na-A) - 374*(angleH-Na-S) - 66924629*(distancePNS-LP)
0.49	0.79	6036	6055235	1978	9	0.71	VOL = 6.45*(distanceNa-ANS)*(angleS-Na-A) - 687*(distancePNS-LP)
0.30	0.61	6231	8315574	2028	7	0.72	VOL = 27339 - 13.63*(distancePNS-LP)*(angleH-Na-S)
0.27	0.57	6969	8709362	2081	4	0.74	VOL = 250615/(distancePNS-LP)

Experiment 7

R^2 Goodness of Fit	Correlation Coefficient	Maximum Error	Mean Squared Error	Mean Absolute Error	Complexity	Fit	Solution
0.70	0.86	4094	3587847	1359	21	0.49	VOL = 385*(distancePNS-VSP) + 7.31*(distanceNa-ANS)*(angleS-Na-A) - 427*(angleH-Na-S) - 0.73*(distancePNS-LP)^3
0.61	0.83	4867	4618997	1549	11	0.55	VOL = 7.24*(distanceNa-ANS)*(angleS-Na-A) - 15.31*(distancePNS-LP)*(angleH-Na-S)
0.44	0.68	5427	6683090	1750	7	0.63	VOL = -20.16*(distancePNS-VSP)*((distancePNS-LP) - 37.02)
0.30	0.57	6540	8261943	2015	4	0.72	VOL = 243823/(distancePNS-LP)

Experiment 8

R^2 Goodness of Fit	Correlation Coefficient	Maximum Error	Mean Squared Error	Mean Absolute Error	Complexity	Fit	Solution
0.67	0.84	5759	3860003	1183	23	0.42	VOL = 142*(angleS-Na-A) + 6.86*(distanceMe-H)*(anglerOr-rPo-Me-rGo) + 4.22*(distancePNS-VSP)*(angleS-Na-A) - 1685 - 16.57*(distancePNS-LP)*(angleH-Na-S)
0.65	0.82	5592	4196326	1238	13	0.44	VOL = 6982 + 3.58*distanceBEPA*(angleS-Na-A) - 17.32*(distancePNS-LP)*(angleH-Na-S)

0.68	0.84	4726	3736811	1247	11	0.45	VOL = 4.58*distanceBEPA*(angleS-Na-A) - 17.73*(distancePNS-LP)*(angleH-Na-S)
0.42	0.79	6317	6903761	1744	9	0.62	VOL = 4.56*distanceBEPA*(angleS-Na-A) - 962*(distancePNS-LP)
0.34	0.61	6549	7774770	1876	7	0.67	VOL = 26553 - 12.1*(distancePNS-LP)*(angleH-Na-S)
0.30	0.62	7051	8314175	2021	5	0.72	VOL = 27528 - 734*(distancePNS-LP)
0.25	0.57	7077	8863189	2111	4	0.75	VOL = 252328/(distancePNS-LP)
0.07	0.75	6893	10994172	2394	3	0.86	VOL = 166*(angleS-Na-A)

Experiment 9

R^2 Goodness of Fit	Correlation Coefficient	Maximum Error	Mean Squared Error	Mean Absolute Error	Complexity	Fit	Solution
0.73	0.87	4582	3175009	1189	23	0.42	VOL = 9.55*distanceBEPA*(distancePNS-VSP) + 6.06*(distanceNa-ANS)*(angleS-Na-A) - 395*(angleH-Na-S) - 0.31*distanceBEPA*(distancePNS-LP)*(distancePNS-VSP)
0.74	0.86	4147	3046593	1243	21	0.44	VOL = 282*(angleS-Na-A) + 11*distanceBEPA*(distancePNS-VSP) - 441*(angleH-Na-S) - 0.306*distanceBEPA*(distancePNS-LP)*(distancePNS-VSP)
0.72	0.86	4554	3342330	1294	19	0.46	VOL = 740*(distancePNS-VSP) + 7.5*(distanceNa-ANS)*(angleS-Na-A) - 476*(angleH-Na-S) - 24.3*(distancePNS-LP)*(distancePNS-VSP)
0.68	0.84	4621	3749244	1321	11	0.47	VOL = 4.62*distanceBEPA*(angleS-Na-A) - 18.1*(distancePNS-LP)*(angleH-Na-S)
0.57	0.82	5407	5085265	1801	9	0.64	VOL = 349*(angleS-Na-A) - 14.3*(distancePNS-LP)*(angleH-Na-S)
0.33	0.62	6790	7976595	1932	5	0.69	VOL = 26720 - 699*(distancePNS-LP)
0.25	0.57	7062	8841476	2107	4	0.75	VOL = 252097/(distancePNS-LP)
0.08	0.75	6868	10915905	2387	3	0.85	VOL = 166*(angleS-Na-A)

Experiment 10

R^2 Goodness of Fit	Correlation Coefficient	Maximum Error	Mean Squared Error	Mean Absolute Error	Complexity	Fit	Solution
0.70	0.86	4380	3512579	1142	15	0.41	VOL = distanceBEPA*(angleS-Na-A)*log((angleS-Na-A)) - 16.55*(distancePNS-LP)*(angleH-Na-S)
0.68	0.84	4706	3759552	1268	11	0.45	VOL = 4.62*distanceBEPA*(angleS-Na-A) - 18.02*(distancePNS-LP)*(angleH-Na-S)
0.40	0.79	6413	7084632	1753	9	0.63	VOL = 4.62*distanceBEPA*(angleS-Na-A) - 981*(distancePNS-LP)
0.34	0.61	6519	7813558	1935	7	0.69	VOL = 25552 - 11.1*(distancePNS-LP)*(angleH-Na-S)
0.27	0.57	6888	8605652	2058	4	0.74	VOL = 249341/(distancePNS-LP)
0.07	0.75	6911	11051944	2400	3	0.86	VOL = 166*(angleS-Na-A)
